# Not All EVs Are Equal: Functional Heterogeneity of Extracellular Vesicles in Neuroregenerative Therapies

**DOI:** 10.3390/ijms27219731

**Published:** 2026-10-30

**Authors:** Magdalena Chodkowska-Michalowska, Oliwia Hawryluk, Szymon Lakomy, Anna Slysz, Anna Sarnowska

**Affiliations:** Translational Platform for Regenerative Medicine, Mossakowski Medical Research Institute, Polish Academy of Sciences, Pawinskiego 5 Street, 02-106 Warsaw, Poland; mchodkowska@imdik.pan.pl (M.C.-M.); ohawryluk@imdik.pan.pl (O.H.); slakomy@imdik.pan.pl (S.L.); aslysz@imdik.pan.pl (A.S.)

**Keywords:** extracellular vesicles, EV heterogeneity, neuroregeneration, stem cell-derived EVs, extracellular vesicle cargo, regenerative medicine

## Abstract

Extracellular vesicles (EVs) are increasingly being investigated as cell-free therapies for neurological diseases. However, their clinical application remains limited by biological and functional heterogeneity. EVs should therefore not be considered as a single, uniform therapeutic product defined only by their cell of origin, size, or conventional vesicle classification. EV composition and activity are influenced by extrinsic factors, including donor and tissue source, culture conditions, and isolation procedures, as well as by intrinsic mechanisms of vesicle biogenesis and selective cargo loading. Some vesicles may have beneficial therapeutic effects, whereas others may show little biological activity or act on unintended cellular targets. For this reason, EVs may be better classified according to biological function rather than only physical properties or cellular origin. A functionality-based classification could improve potency assessment, dose selection, reproducibility, and the development of consistent EV-based products. EV properties can be modulated by selecting appropriate parental cells, modifying culture conditions, or using genetic or metabolic engineering to alter EV production and cargo. Single-vesicle approaches can reveal differences between EV subpopulations and identify those responsible for specific biological effects. Overall, understanding EV heterogeneity may provide a rational basis for developing reproducible and disease-specific EV therapies for neurological and neurodegenerative disorders.

## 1. Introduction

Extracellular vesicles (EVs) are a heterogeneous population of membrane-bound particles released by all cell types under physiological and pathological conditions. Acting as key mediators of intercellular communication, EVs transport a broad range of bioactive cargo, including proteins, lipids, metabolites, DNA, messenger RNA, and non-coding RNAs, thereby influencing the behavior and function of recipient cells. In earlier studies, extracellular vesicles (EVs) were often categorized according to their presumed biogenesis. However, current guidelines discourage the routine use of biogenesis-based terminology unless the vesicular origin has been directly demonstrated. Instead, EVs are now preferentially described using operational criteria, most commonly size, for example as small EVs (<200 nm) and large EVs (>200 nm) [1].

The growing interest in EV-based therapeutics, particularly those derived from mesenchymal stromal/stem cells (MSCs), stems from their ability to possess most of the beneficial effects of their parental cells while avoiding several limitations associated with cell transplantation. Unlike living cells, EVs lack self-replicative capacity, reducing the risk of uncontrolled proliferation or tumor formation. Furthermore, their relatively low immunogenicity, together with improved stability, storage, and transport characteristics, makes them attractive candidates for cell-free regenerative therapies [2,3,4] (Table 1). Importantly, EVs can be derived not only from stem cells, but also from a wide range of other cell types, including human amniotic epithelial cells, immortalized cerebral endothelial cells, macrophages, dendritic cells, astrocytes, and human umbilical cord blood-derived mononuclear cells [5,6,7,8,9,10].

Indeed, EVs are now widely recognized as principal mediators of the paracrine mechanisms underlying many of the therapeutic benefits previously attributed to stem cell transplantation, which has contributed to their emergence as a rapidly growing field of biomedical research in neurological disorders, including traumatic brain injury (TBI), spinal cord injury (SCI), amyotrophic lateral sclerosis (ALS), Alzheimer’s disease (AD), and Parkinson’s disease (PD) [5,7,13,19,28,29,30]. This is related to their potential use in neuroregenerative therapies, which aims to support the regeneration, repair and/or restoration of function in damaged nervous tissue. In the case of cells and vesicles, such therapy may involve indirect (immunomodulatory/paracrine) effects, whereas in the case of cell therapies, it may also involve direct (cell replacement) effects. The expanding therapeutic application of EVs is not confined to neurology; nevertheless, neurological disorders provide a useful framework for highlighting broader challenges and conceptual shortcomings that are relevant to EV-based therapies across multiple medical disciplines.

According to data available as of May 2026, the International Clinical Trials Registry Platform (ICTRP) lists 4586 clinical trials involving stem cell-based therapies across various phases of clinical development. Despite this extensive research activity, only three stem cell-based therapies have received marketing authorization from the European Medicines Agency (EMA): *Holoclar*, *Zemcelpro,* and *Alofisel*, although the latter was subsequently withdrawn at the request of the marketing authorization holder [31].

In comparison, the same database lists 1527 EV-based therapeutic approaches; however, none has yet received EMA marketing authorization.

These observations raise several important translational questions.

1. Why has the field not shifted entirely from cell-based approaches toward EV-based therapeutics if EVs are considered safer and more controllable alternatives to stem cell-based therapies?
2. Why do EV-based therapies still fail to produce robust and reproducible therapeutic outcomes despite the rapidly growing number of preclinical studies?
3. Why do EVs derived from the same parental cell source often yield markedly different biological effects across studies?

In this context, an important question emerges: are the current limitations of EV-based therapies fundamentally different from those previously observed in stem cell-based approaches, or are we simply rediscovering the same translational challenges at the extracellular vesicle level?

## 2. “Is It Realistic from the Biological Point of View to Expect That Non-Modified EV-Based Therapy Could Effectively Treat Different Neurological Disorders?” Similar Pathology, Different Biology

In many cases, therapeutically distinct neurological disorders characterized by different underlying pathological mechanisms are approached using naive, non-modified EV-based strategies. Consequently, the same undefined mixture of EV-associated molecules is often expected to modulate mechanistically distinct disease processes.

Although many neurological disorders share certain pathological features, including dysregulated neuroinflammation, oxidative stress, mitochondrial dysfunction, and progressive neuronal degeneration [5,7,13,19,28,29,30], the molecular mechanisms driving these processes differ substantially between diseases and similar pathological outcomes do not necessarily reflect identical disease biology. Depending on the pathological context, neuroinflammation may arise from protein aggregation, mitochondrial dysfunction, oxidative stress, blood–brain barrier disruption, excitotoxicity, or excessive activation of innate and adaptive immune responses [19,30,32,33]. Under physiological conditions, neuroinflammation represents a tightly regulated protective response aimed at restoring tissue homeostasis and promoting tissue repair [28]. However, prolonged or uncontrolled activation of microglia and astrocytes may shift this process toward a chronic pathological state characterized by excessive cytokine production, reactive oxygen species (ROS) accumulation, mitochondrial dysfunction, and progressive neuronal damage [34,35]. ROS represent one of the major mediators linking neuroinflammation with neurodegeneration. Although ROS participate in physiological signaling and host-defense responses, excessive ROS production or impaired antioxidant defense leads to oxidative stress, oxidation of cellular components, mitochondrial dysfunction, and ultimately neuronal cell death [32,35]. Importantly, oxidative damage further amplifies neuroinflammation through the release of pro-inflammatory mediators from injured cells, thereby creating a self-propagating cycle of inflammation and neurodegeneration [14].

In neurodegenerative disorders such as Alzheimer’s disease and Parkinson’s disease, chronic neuroinflammation is closely associated with toxic protein accumulation, oxidative stress, and progressive neuronal degeneration [13,19,36,37]. In contrast, in traumatic brain injury (TBI) and spinal cord injury (SCI), neuroinflammation initially plays a protective role by supporting debris clearance and tissue remodeling; however, excessive or persistent immune activation frequently exacerbates tissue damage and limits regenerative capacity [30,37,38]. Similarly, in amyotrophic lateral sclerosis (ALS), chronic activation of inflammatory signaling pathways contributes not only to motor neuron degeneration, but also to impaired skeletal muscle regeneration through dysregulated macrophage polarization and NF-κB signaling [39,40,41].

Together, these observations indicate that neuroinflammation represents a shared pathological outcome rather than a uniform disease mechanism, highlighting the need for disease-specific therapeutic strategies rather than universally applied, non-modified EV-based approaches. Although oxidative stress and neurodegeneration are commonly observed across neurological disorders, the molecular mechanisms driving these processes vary considerably between diseases.

The mechanistic diversity underlying apparently similar pathological outcomes can be observed across multiple neurological disorders. In Parkinson’s disease (PD), oxidative stress and neurodegeneration are closely associated with progressive degeneration of dopaminergic neurons, chronic microglial activation, and reduced activity of endogenous antioxidant enzymes such as catalase and superoxide dismutase [10,36,42,43,44,45]. In contrast, hereditary forms of amyotrophic lateral sclerosis (ALS) are associated with mutations in the SOD1 gene, resulting in impaired superoxide detoxification, mitochondrial dysfunction, and progressive motor neuron degeneration [46,47,48,49]. Meanwhile, traumatic brain injury (TBI) and ischemic injury are primarily driven by oxygen and nutrient deprivation, ATP depletion, secondary ROS overproduction, and mitochondrial dysfunction following tissue damage [5,7,50,51]. Furthermore, the regenerative capacity of injured brain tissue remains limited due to vascular damage, glial scar formation, and restricted neuronal regeneration [16].

Since neurological disorders are driven by distinct pathological mechanisms, EV-based therapies should target specific disease-related pathways rather than be expected to act through one universal mechanism.

### Different Diseases Require Different EV Functions

One of the best-described therapeutic functions of EVs is the modulation of dysregulated neuroinflammation. EVs are able to transfer biologically active molecules, including miRNAs, proteins, cytokines, and antioxidative enzymes, which can alter inflammatory signaling pathways and immune-cell behavior. In mouse models of traumatic brain injury (TBI), EVs derived from astrocytes stimulated with post-traumatic brain extracts promoted the transition of microglia from the pro-inflammatory M1 phenotype toward the anti-inflammatory M2 phenotype [5]. This effect was associated with miR-873a-5p-mediated inhibition of the NF-κB pathway, leading to reduced neuroinflammation, decreased brain edema, and smaller injury area [5].

Similar immunomodulatory effects were observed in amyotrophic lateral sclerosis (ALS). EVs derived from regenerating muscle (RMEVs) promoted macrophage polarization toward the anti-inflammatory M2 phenotype, reduced NF-κB activation, and enhanced muscle regeneration and functional recovery [40]. Additionally, these EVs increased expression of anti-inflammatory mediators such as IL-10, Arg1, and Chil3 [40]. In another ALS study, mesenchymal stem cell-derived EVs (MSC-EVs) reduced oxidative stress and inflammatory signaling in motor neurons, resulting in decreased motoneuron degeneration, demyelination, and synaptic denervation, together with improved grip strength, survival, and motor performance in ALS mouse models [48]. In spinal cord injury (SCI), EVs secreted by regulatory T cells inhibited microglial pyroptosis, most likely through delivery of miR-709, leading to reduced expression of inflammatory mediators such as NLRP3 and IL-1β and improved motor recovery [38].

Besides their immunomodulatory activity, many EV populations also exert antioxidant and neuroprotective effects. In Parkinson’s disease (PD), EVs enriched with miR-124-3p reduced oxidative stress and apoptosis while protecting dopaminergic neurons from degeneration [10]. These effects were associated with modulation of signaling pathways involved in autophagy and neuronal survival, including AMPK/mTOR and p62/p38 signaling [10]. Importantly, treatment with miR-124-3p-enriched EVs improved motor function in PD mouse models [10]. Similarly, catalase-loaded EVs effectively reduced ROS accumulation in the substantia nigra after intranasal administration, decreasing activation of microglia and astrocytes and protecting dopaminergic neurons from degeneration [14].

Enhanced antioxidant activity has also been achieved using engineered EVs. In TBI models, selenium nanoparticle-coated EVs (SeEVs) demonstrated higher ROS-scavenging capacity than free selenium or unmodified EVs [17]. Treatment with SeEVs reduced oxidative stress, inflammation, neuronal apoptosis, and improved behavioral recovery following brain injury [17].

Another important therapeutic mechanism involves restoration of metabolic homeostasis and mitochondrial function. EVs carrying mitochondria (MitEVs) increased oxygen consumption rate, ATP production, glycolytic capacity, and cell survival under hypoxic conditions [7]. MitEV treatment also increased mitochondrial content in cortical and hippocampal neurons, suggesting a potential role in disorders associated with energy depletion and mitochondrial dysfunction [7].

EVs may also support neuronal regeneration and tissue remodeling after CNS injury. In TBI models, MSC-derived EVs embedded within hyaluronic acid–collagen hydrogels promoted neuronal differentiation, axonal outgrowth, synapse formation, remyelination, angiogenesis, and vascular maturation [16]. At the same time, these EVs reduced astrocytic differentiation and glial scar formation, thereby improving regenerative processes within injured brain tissue [16].

Increasingly, EV engineering strategies are being developed to improve disease-specific therapeutic efficacy. In PD, dopamine-modified EVs (DopaEVs) exhibited enhanced uptake by recipient cells and reduced dopaminergic neurodegeneration and α-synuclein toxicity, mainly through autophagy-related mechanisms [42]. In Alzheimer’s disease (AD), EVs have been used as targeted delivery systems for therapeutic cargo directed against amyloid pathology. EVs carrying siRNA against BACE1 reduced β-amyloid production following targeted brain delivery [13]. Similarly, Fe65-modified EVs carrying Corynoxine-B enhanced autophagy and reduced APP, Aβ, and amyloid plaque accumulation in AD mouse models [19]. More advanced approaches include MAPLEVs, in which photoinduced release of CRISPR-dCas9 complexes enabled targeted silencing of the Bace1 gene and reduced amyloid pathology in AD models [15].

Together, these studies indicate that EV populations may differ substantially in their biological activity depending on their cellular origin, cargo composition, and engineering strategy. Notably, many of the most effective therapeutic approaches rely on engineered or functionally modified EVs rather than unmodified EV populations. This further supports the idea that EV heterogeneity [52] is not only a technical issue, but also a biologically important feature that may determine therapeutic efficacy in different neurological disorders [52].

## 3. If Different Neurological Disorders Require Distinct Therapeutic Strategies, Another Important Question Emerges: Which Extrinsic Factors, Including Cellular Origin and Environmental Cues, Determine the Therapeutic Phenotype of EV Populations and Their Suitability for Specific Clinical Applications?

This disease-level diversity provides a first reason to question the concept of a universal EV product: if different disorders require distinct mechanisms of action, the therapeutic relevance of EV heterogeneity extends beyond descriptive characterization to the selection of EV populations suited to specific clinical contexts.

Far from being standardized biological products, EVs are highly responsive to external influences, with donor-cell origin, culture conditions, and isolation procedures profoundly affecting their molecular composition and therapeutic activity.

### 3.1. Source

One of the major factors influencing EV heterogeneity is the tissue source. EVs derived from MSCs isolated from bone marrow, adipose tissue, dermal tissue, dental pulp, and umbilical cord exhibit similar size distribution, morphology, and zeta potential, as well as comparable levels of protein and mRNAs [53]; however, slight, statistically insignificant differences in vesicle diameter were observed [53]. The largest vesicles isolated from UCSC and DPSC, measuring approximately 160 nm. The mean diameters of EVs derived from BMSCs, DSCs, and ASCs were approximately 140, 135, and 130 nm, respectively [53]. It is worth noting that the highest number of EVs measured by NTA was obtained from cells isolated from the umbilical cord, whereas the number of EVs was similar among cells from other tissues [53]. This observation was replicated in the protein extracted from the EVs [53]. Regardless of storage temperature or transport mode, EVs derived from MSCs of different tissue origins demonstrated comparable stability. The analysis of the vesicles revealed that EVs from different sources share 883 proteins out of a total of 1696 detected proteins [53]. The shared proteins are associated with metabolism, human diseases, cellular processes, environmental information processing, and organismal systems, highlighting the extensive involvement of EVs in cellular homeostasis and intercellular communication [31]. The characterization of EVs derived from BM-MSCs revealed an increased number of metabolic proteins, while those derived from UCSC-MSCs exhibited immune response characteristics. Furthermore, the analysis of ASC-EVs demonstrated cardiovascular biosynthesis and glycan metabolism, in addition to cell growth and death. In addition, dermal EVs were found to be associated with infectious diseases, and dental pulp EVs were identified as markers of the nervous system, more specifically, with neurological processes. It is noteworthy that EVs derived from MSCs obtained from UC and DP exhibited significantly higher BM loading efficiency in comparison to those derived from dermal tissue [53].

The next factor is cell line status. The research also investigated the disparities between EVs derived from primary and immortalized WJ-MSCs. Both kinds of EVs share 65 miRNAs. Furthermore, there is no statistically significant difference in nitric oxide (NO) secretion between the two types of EVs. Moreover, no apparent difference was observed when using both types of EVs for wound healing [54].

The donor is also a factor influencing the variability of EV. Studies have shown that EVs derived from different adipose-derived stem cell (ASC) donors exhibit significant differences in their lipid profiles (free fatty acids, glycerides, glycerophospholipids, sphingolipids, and sterols) [55].

### 3.2. Culture Conditions

The heterogeneity of EVs is influenced also by MSC culture conditions, such as the composition of the culture medium and the oxygen concentration. It is imperative to acknowledge the pivotal role of protein supplementation in the culture medium, whether through the incorporation of FBS or hPL, on the secretory characteristics of MSCs. The number of EVs released by MSCs in cultures supplemented with FBS decreases as the passage number increases. No such differences were observed in cultures supplemented with human platelet lysate. No differences were observed in the diameter or zeta potential of MSC-EVs for either supplement. Furthermore, no differences were observed in the expression profiles of angiogenesis mRNAs and miRNAs when using FBS or hPL [56].

An important factor is the amount of oxygen in cell culture. No differences in EV size were observed between hUC-MSCs cultured under hypoxic or normoxic conditions [57,58]. However, EVs derived from cells cultured under normoxic conditions exhibit higher levels of inflammation score and a higher PAS score than those obtained from cells cultured under hypoxic conditions. Furthermore, MSCs cultured under hypoxic conditions secrete a statistically significantly higher number of EVs than those cultured under normoxic conditions. The researchers also observed increased expression of miR-146a in vesicles derived from hypoxic cells compared to normoxic cells [58].

### 3.3. Method of Isolation

The heterogeneity of the vesicles may also be influenced by the EV isolation method. Using the precipitation method to isolate EVs proved to be much more effective in terms of protein yield and vesicle count for EVs derived from chorionic tissue-derived mesenchymal stromal cells than the precipitation followed by ultracentrifugation method or size exclusion chromatography (SEC). The size of the EVs was similar across all methods. Notably, EVs isolated by precipitation exhibited increased TMP1 expression compared to those isolated using the other methods. The differences between the isolation methods for the remaining factors (MPP1, MMP3, COX2, TSG-6, COL10A, ACAN and COL2A) were not statistically significant [59].

EV heterogeneity results not only from technical and environmental factors, such as cell source, culture conditions, and isolation methods, but also from intrinsic biological mechanisms including vesicle biogenesis and selective cargo loading.

These factors are well recognized in the context of cell-based therapies. However, despite efforts to standardize them, a much greater challenge lies in the intrinsic heterogeneity of EV populations.

## 4. Biogenesis as a Source of Intrinsic EV Heterogeneity

Although EVs are often discussed collectively, they do not represent a uniform biological entity. Instead, EV populations comprise multiple vesicle subtypes that differ in size, biogenesis route, molecular cargo, surface composition, density, uptake capacity, biodistribution, and biological activity [1,60,61]. As a result, EV preparations isolated even from the same source do not represent fully homogeneous products, but rather mixtures of vesicle subpopulations with distinct molecular and functional properties. This intrinsic heterogeneity means that individual vesicles within a bulk EV preparation may not share all chemical or physical characteristics of the total population, and only selected EV subpopulations are likely to mediate specific biological effects [60,62]. Consequently, from a therapeutic perspective, an important question is not only which cell type produced the EVs, but also which EV subpopulations are biologically active, functionally neutral, or potentially antagonistic toward the intended therapeutic response [63,64].

One of the major drivers of EV heterogeneity is EV biogenesis. Biogenesis is not a passive or random process, but rather a tightly regulated and context-dependent sequence of events that determines what type of vesicles a cell produces, which molecules are loaded into them, and consequently what biological functions they may perform [65]. Different biogenesis pathways impose distinct molecular constraints on vesicle formation, thereby influencing membrane composition, cargo selection, and functional potential. Endosomal pathways generate small EVs enriched in tetraspanins and regulatory RNAs, whereas plasma membrane budding produces microvesicles containing cytoskeletal proteins and signaling receptors. Additional mechanisms, such as mitochondrial extrusion [66] and secretory autophagy [67], further expand EV heterogeneity.

Essentially, cargo loading into EVs is not random. Proteins, lipids, and RNAs are actively sorted into EVs through mechanisms involving ESCRT complexes, tetraspanins, lipid microdomains, and RNA-binding proteins. Because these molecular machineries dynamically respond to environmental and pathological stimuli, EVs produced under different physiological or pathological conditions may differ substantially in their molecular profiles and biological activity [62,68]. Thus, biogenesis directly links EV formation with EV function and is also a major target for EV engineering strategies, since modification of the machinery responsible for vesicle formation or cargo loading may alter EV composition and therapeutic properties.

The biogenesis of EVs is therefore one of the central determinants of EV heterogeneity. Cells employ multiple mechanistically distinct pathways to generate vesicles, each producing EV subpopulations with unique molecular signatures and biological functions. Multiple intraluminal vesicle (ILV)-forming mechanisms contribute to exosome heterogeneity. In ESCRT-dependent pathways, the ESCRT machinery (ESCRT-0, I, II, and III) drives membrane invagination and sorting of ubiquitinated proteins [69]. ESCRT-associated proteins such as ALIX and TSG101 are important regulators of this process and are commonly used as exosomal markers [70].

ESCRT-independent pathways include ceramide-dependent budding driven by neutral sphingomyelinase, phospholipase D2 (PLD2)-dependent pathways, and tetraspanin-mediated cargo sorting involving CD9, CD63, CD81, and CD82. Significantly, tetraspanins participate in cargo selection through both ESCRT-dependent and ESCRT-independent mechanisms, reinforcing their role in generating distinct exosome subpopulations [71].

In contrast to exosomes, ectosomes (also referred to as microvesicles or shedding vesicles) are generated by outward budding of the plasma membrane. This mechanism differs fundamentally from endosomal ILV formation and produces vesicles that more closely reflect the molecular composition of the plasma membrane [62].

Importantly, intrinsic heterogeneity extends beyond the mechanisms of vesicle biogenesis. Distinct EV subpopulations can coexist within the same preparation and differ in their physical properties, molecular composition, and biological activity, making conventional labels such as exosomes or microvesicles insufficient to define their functional identity.

## 5. EV Heterogeneity and the Limits of Source-Based Classification

**Figure 1:**
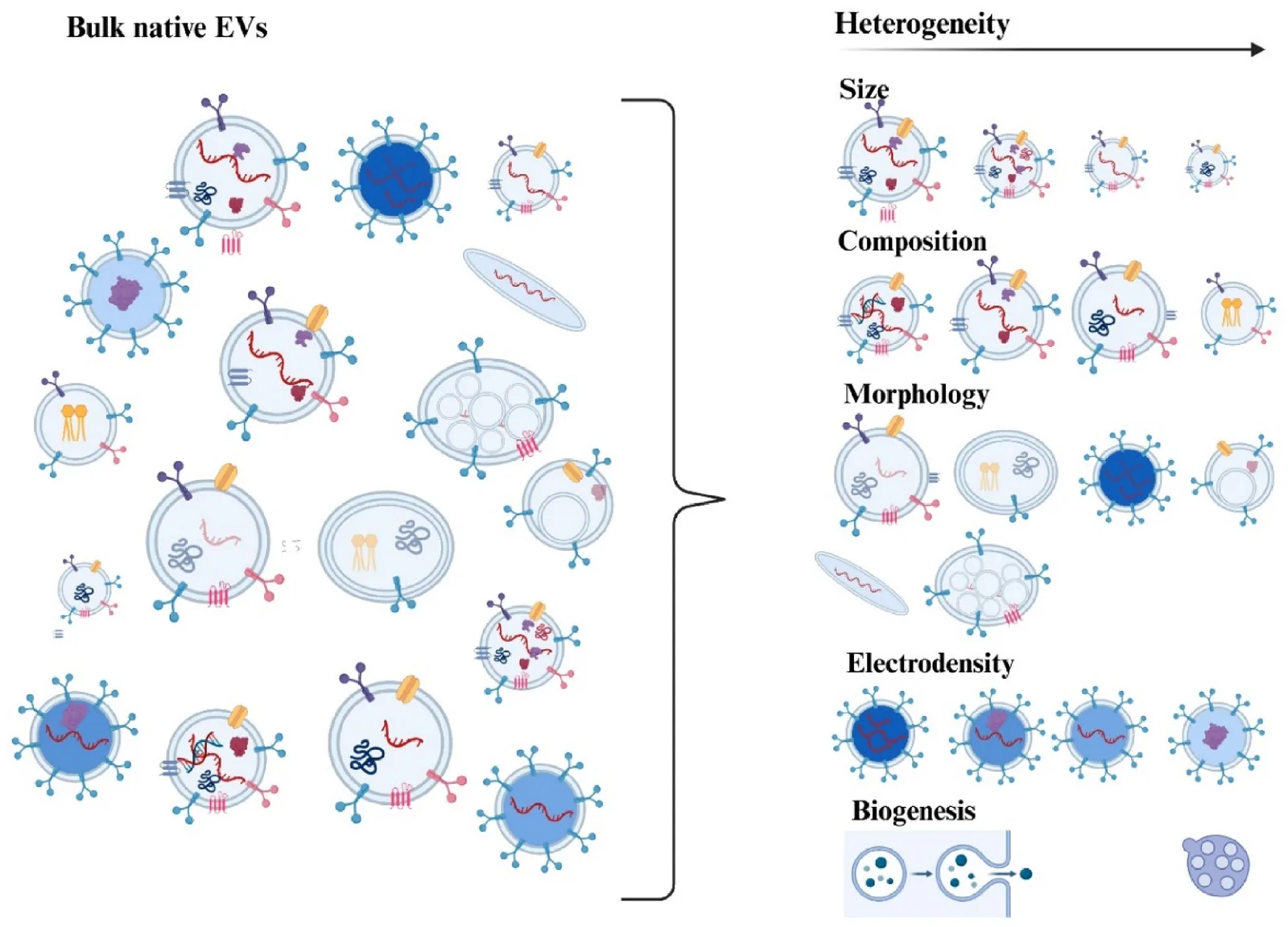
Multilevel heterogeneity of EV. Bulk native EVs display heterogeneity in size, composition, morphology, electrodensity, and biogenesis, highlighting the complexity of EV populations. This figure was prepared based on the concept presented by Carney [60].

EVs represent a non-homogeneous set of vesicles in terms of biogenesis, origin, density, size, morphology, and cargo composition (Figure 1) [72,73]. Traditional classification into exosomes, microvesicles, and apoptotic bodies is useful as a conceptual framework, but it has important limitations [74]. The size ranges of different EV classes overlap, isolation methods do not yield pure populations, and vesicles with similar physical parameters may differ markedly in surface markers and biological function [73]. This is why current EV nomenclature increasingly emphasizes operational definitions and transparent characterization rather than assuming that a given isolation method produces one biologically uniform vesicle class [72].

Morphological studies further support this view. EVs were historically often described as simple spherical particles using electron microscopy, but cryo-electron microscopy (Cryo-TEM) has shown that EV morphology is much more diverse [75,76]. In near native conditions, vesicles may appear as single vesicles, double membrane structures, multilamellar vesicles, vesicles containing smaller vesicles, tubular structures, or particles with different electron densities [76,77]. Zabeo et al. reported substantial morphological variability among EVs isolated from a single cell type, including differences in shape, size, and luminal density, suggesting that even one parental cell population can release multiple EV subtypes [78]. Similar observations in minimally processed biological fluids support the idea that EV diversity is not merely an artefact of isolation, fixation, or staining procedures [76].

This diversity has functional implications. If a therapeutic EV preparation contains multiple vesicle subtypes, the observed biological effect represents a net outcome of all vesicles present in the product [72,79]. Some subpopulations may carry active therapeutic cargo and be efficiently internalized by target cells, while others may be biologically neutral, poorly internalized, or diverted to off-target cells [63,80]. In some cases, vesicles may even compete for uptake, bind receptors without inducing the intended response, or dilute the activity of the truly functional fraction [72,73]. Therefore, heterogeneity can be both an advantage and a disadvantage. It provides biological complexity and a wide repertoire of signals, but it also complicates reproducibility, potency assessment, and clinical translation [72,81].

Taken together, these observations shift the central question from whether an EV preparation is heterogeneous to how that heterogeneity contributes to the final therapeutic effect.

The functional consequences of this heterogeneity become particularly apparent after administration. The route of EV administration can significantly affect their biodistribution, retention in tissues, and the functional heterogeneity of the vesicle populations reaching various organs. Studies indicate that different routes of administration result in distinct distribution profiles of EVs (derived from human bone marrow MSCs) within the body. After intravenous administration, EVs are largely taken up by peripheral organs, primarily the liver and spleen, whereas after intraperitoneal injection, their increased presence was observed, among others, in the pancreas and the gastrointestinal tract, and following subcutaneous administration, mainly within the gastrointestinal tract [82]. A similar pattern was observed in other studies, in which, following intravenous and intraperitoneal administration, the largest proportion of EVs (derived from human amniotic MSCs) was detected in the liver, while only small amounts of vesicles were present in the brain, located, among other areas, in the motor cortex (M), somatosensory cortex (S) and caudoputamen (CP) regions [83]. Following intravenous administration, EVs (derived from anaplastic thyroid cancer cell—Cal62) were also detected in the lungs, heart, liver, spleen, and kidneys, although the signal in these organs decreased over time, which may be related to their elimination from the body, including via urine [84].

Other studies also show that, following intravenous administration, a strong EV (derived from Expi293F) signal was observed in the liver and spleen, as well as some uptake in the lungs, whereas no comparable signal was observed in the lungs following intranasal administration. The uptake of EVs by the kidneys, heart, large intestine, and brain was limited regardless of the route of administration. At the same time, following intravenous administration, a weak signal was detected in the medulla oblongata, which may indicate that this area is more accessible to EVs present in the circulation, although the signal level was significantly lower than in the liver and spleen [85]. In contrast, a study comparing intravenous, intrapleural, and intratracheal administration showed that, following intravenous administration, EVs (derived from human Umbilical Cord MSCs) were primarily present within the chest, and the signal gradually decreased over 72 h. Intrapleural administration was characterized by the highest signal intensity and the longest retention time, whereas after tracheal administration, EVs exhibited a more uniform distribution within the chest and were present primarily in the lungs. Organ analysis after 72 h also showed that EVs administered intrapleurally and intravenously accumulated mainly in the liver, whereas those administered into the trachea were predominantly found in the lungs [86].

The above observations indicate that the route of administration may determine not only the amount of EVs reaching specific tissues, but also their availability to specific cell populations and, consequently, their potential biological effect. Differences in EV biodistribution resulting from both their origin and the route of administration used underscore the need to optimize delivery methods and select an appropriate source of EVs depending on the target tissue. At the same time, genetic and chemical modifications of EVs can enhance their targeting of specific organs, potentially limiting the rapid clearance and high retention of native EVs in organs such as the liver. Alternative strategies, including the use of plant- or milk-derived EVs, EV mimetics, synthetic vesicles, and the combination of EVs with advanced biomaterials, may further enhance control over their release, retention in tissues, and delivery efficiency [87]. In addition, compared to larger microvesicles, EVs show greater potential for enhancing cellular and mitochondrial bioenergetics in endothelial cells lining the blood–brain barrier (BBB) and in neurons [7].

The intranasal route is a particularly interesting alternative to intravenous administration. It may allow for a more direct delivery of EVs to the brain, potentially partially bypassing the blood–brain barrier, and thus increase their availability in the CNS. Furthermore, intranasal administration may limit exposure of peripheral organs and can be used to deliver EVs to other tissues, including the lungs, as well as to modulate the immune response of mucosal membranes. For this reason, the intranasal route is increasingly being considered as a potentially beneficial strategy for delivering EVs in therapies for neurological and respiratory diseases [88].

Consequently, different routes of administration may lead to the formation of functionally distinct populations of EVs in terms of their biodistribution, stability, ability to cross biological barriers, retention in tissues, and affinity for specific cells. For this reason, the biological effects of EVs in the CNS should be considered not only in terms of their source and molecular composition, but also in relation to the route of administration used, which may determine which functional fraction of EVs ultimately reaches the target tissue.

**Figure 2:**
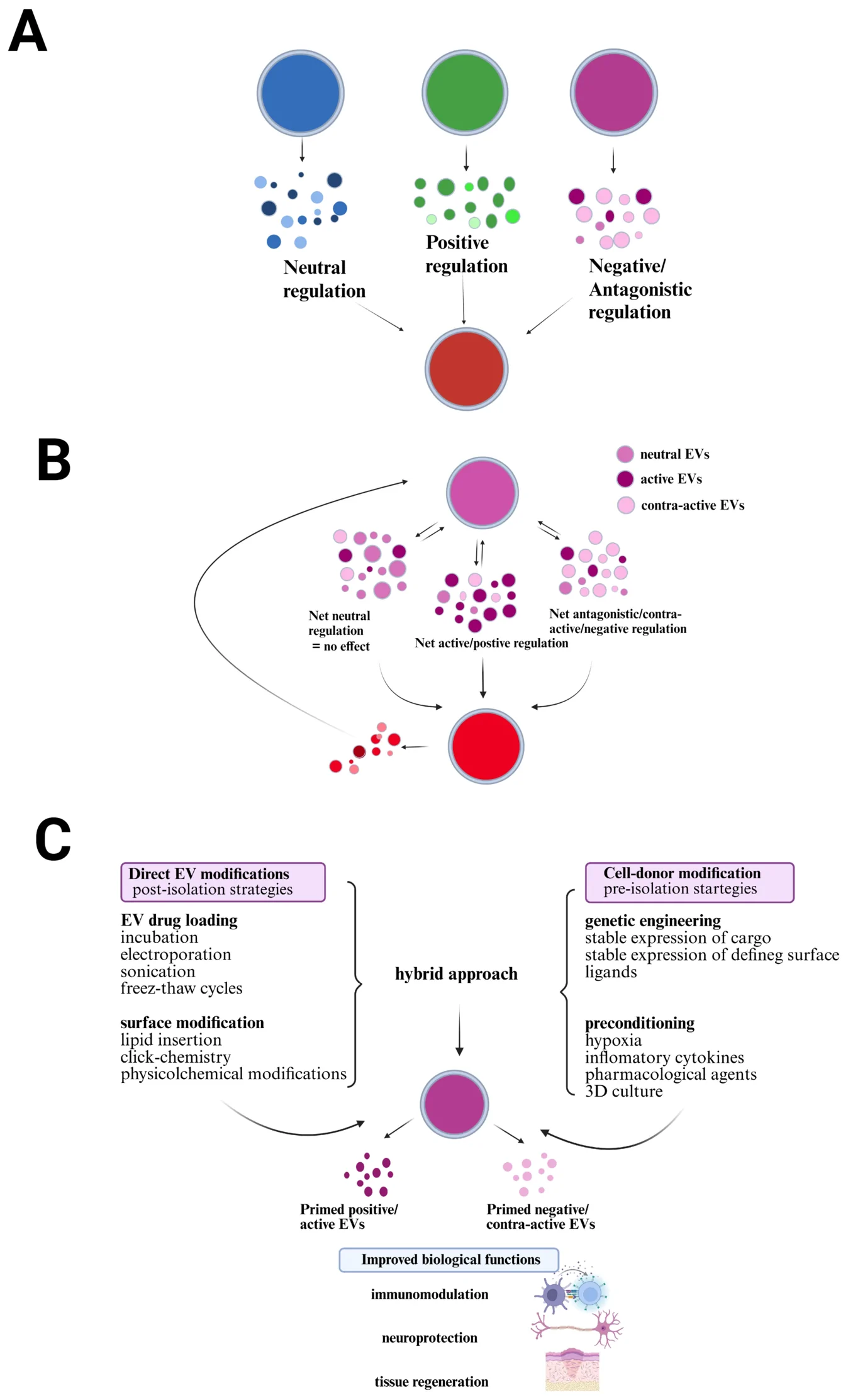
A conceptual framework for the functional reclassification of MSC-EVs. (**A**) EVs released by a given cell source are functionally heterogeneous. (**B**) The overall biological effect reflects the net outcome of heterogeneous EV subpopulations. (**C**) Priming can enrich distinct EV subpopulations with desired therapeutic functions. The analytical approach was modeled on the methodology described by [72]. This figure was created using BioRender. Chodkowska, M. (2026). https://BioRender.com/qc1fecu (accessed 22 October 2026).

Some vesicles reach the intended recipient cells and trigger a biological response, some remain functionally silent, and others may be taken up by non-target cells or compete with active vesicles for receptor binding, tissue localization, or endocytic capacity [82,89]. Thus, the biological outcome of a bulk EV preparation reflects the combined effects of multiple co-existing vesicle subpopulations, which may include therapeutically active, functionally neutral, competitive, or decoy-like, and off-target vesicles (Figure 2) [60,72]. This idea is consistent with systems-level interpretations of EV biology, in which the final response depends on emergent properties of heterogeneous EV populations acting together rather than on one isolated cargo molecule or vesicle subtype [90,91].

### 5.1. EV Heterogeneity and the Limits of Conventional Classification

Vesicles with similar size may differ in molecular identity, while vesicles with similar marker expression may differ in functional activity. Similarly, EVs derived from the same donor cell population can contain distinct subtypes with different density, cargo, morphology, and biological effects (Figure 2).

Lässer and colleagues emphasized that the EV secretome is highly diverse and that a single cell type may release multiple EV subtypes differing in density, molecular cargo, and morphology [72]. This means that a label such as MSC-EVs, exosomes, or small EVs does not necessarily define a therapeutically equivalent population [92].

A more useful framework for translational applications may therefore be a functionality-based classification. In this approach, EVs are proposed to not be defined only by their origin or size, but by their contribution to the final biological effect. This is especially relevant for EV-based therapies, where two preparations with similar particle numbers may produce different outcomes because the proportion of active, neutral, and off-target vesicles differs. In this context, EV heterogeneity becomes not only a technical challenge, but also a biological determinant of therapeutic potency [64].

### 5.2. Functional EVs—Vesicles Carrying Active Cargo and Inducing a Biological Response

The first functionally relevant category includes active or functional EVs. These vesicles carry biologically relevant cargo, display appropriate surface molecules, reach the intended recipient cells, and induce the desired effect. In neurological applications, such effects may include immunomodulation, neuroprotection, angiogenesis, axonal regeneration, synaptic support, modulation of microglial activation, or protection of the blood–brain barrier [93]. Functional EVs can act through several mechanisms. Despite the common way of transferring biological value via delivering miRNAs, mRNAs, proteins, lipids, enzymes, or metabolites into recipient cells, EVs may also signal through surface molecules without complete internalization. Some may influence extracellular signaling by binding ligands, matrix components, or receptors [94]. A novel perspective on the mechanisms underlying the activity of extracellular vesicles is provided by the study of Thong et al., in which the authors highlight the possibility that individual EVs influence multiple cells by modulating the cellular microenvironment through the catalytic activity of vesicle membrane-associated enzymes, such as CD73 and CD59 [95]. Importantly, not all vesicles in a bulk EV preparation have this capacity. Only a specific subset may be responsible for the dominant biological response [96]. Experimental evidence supports this concept. Van de Wakker et al. identified three distinct small EV subpopulations from a single source, with different proteomic profiles and different functional effects in cardiac repair assays. Notably, the largest-sized subpopulation showed no effect in the tested functional assays, whereas other fractions contributed more clearly to biological activity [97]. This observation is crucial because it indicates that functional activity may be confined to specific EV subpopulations, whereas other vesicles present in the same preparation may lack the molecular features required to mediate the desired biological effect and thus contribute little or nothing to the overall outcome [60,62].

Similarly, studies comparing EVs released from different cellular domains demonstrated that even one cell type can produce vesicles with various cargo and function depending on their site of release. Wang et al. showed that apical and basolateral EV populations differ in composition and biological properties, supporting the idea that EV identity is shaped not only by donor cell type, but also by vesicle origin and release route [98]. Together, these findings challenge the assumption that EVs preparations constitute homogeneous therapeutic entities.

### 5.3. Neutral EVs—Vesicles Present in the Preparation but Functionally Silent

A second functionally important group can be described as neutral EVs. These vesicles are present in an EV preparation but do not contribute meaningfully to the desired therapeutic effect. They may lack the relevant cargo, display insufficient levels of key surface molecules, fail to bind the target cell, or be internalized without releasing functional cargo [99,100]. From the perspective of bulk quantification, they still increase particle number, protein content, or tetraspanin signal, but from the perspective of therapy, they may dilute the active fraction [101]. The existence of neutral EVs is particularly important because many EV studies still rely heavily on particle number or total protein amount as dose approach [102]. However, if only a subset of vesicles is biologically active, then the total number of particles may poorly reflect the functional dose. A preparation containing a high proportion of neutral vesicles may appear quantitatively rich but functionally weak. Conversely, a lower particle dose enriched in active vesicles may be more potent.

Neutrality may also result from inefficient uptake. The presence of an EV near a cell does not guarantee binding, internalization, endosomal escape, or biological activity [103]. EV uptake is selective and can depend on the donor–recipient cell combination, surface adhesion molecules, membrane composition, and the physiological state of recipient cells [104]. Jurgielewicz et al. used imaging flow cytometry to demonstrate that EV uptake has measurable kinetics and specificity, reinforcing the idea that EV-cell interactions are not random and that not every vesicle can be efficiently internalized by every recipient cell [105]. Thus, a vesicle may be structurally valid as an EV but functionally irrelevant in a given therapeutic context.

Neutral EVs should be understood as functionally inactive in a given biological context, rather than intrinsically defective; the same vesicle fraction may be inactive in one disease model or target cell type yet active in another, depending on the microenvironment and recipient-cell state [68,106].

### 5.4. Competitive, Decoy-like, and Off-Target EVs

A third category includes EVs that may reduce, redirect, or block the desired activity of functional vesicles. Such vesicles can be described as competitive EVs, antagonistic, decoy-like EVs, off-target or functionally counterproductive EVs [101,107]. These vesicles may interfere with therapy in several ways. First, they may compete with active EVs for limited receptor-binding sites on target cells. Recipient cells have finite surface receptor availability and endocytic capacity, so an excess of vesicles without the appropriate cargo could reduce uptake of the therapeutically active fraction. Second, they may bind receptors or ligands without inducing the intended downstream response, effectively blocking or dampening signaling. Third, they may be preferentially taken up by non-target cells, thereby reducing the dose that reaches the therapeutic site. Fourth, they may carry cargo that produces an effect opposite to the intended one, especially in inflammatory or disease-derived EV populations.

The concept of EVs as decoys is biologically plausible and increasingly recognized. A review on EV decoying in host immunity describes EVs as structures capable of modulating immune responses not only through cargo delivery, but also through sequestration of ligands, toxins, pathogen-associated signals, or inflammatory mediators [104]. For instance, Schwann cell-derived EVs containing TNFR1 can act as TNFα decoys, binding TNFα and limiting its availability for cellular signaling [108]. Similarly, engineered or replenished decoy EVs have been used to modulate inflammatory signaling pathways, showing that this mechanism can be therapeutically exploited [107].

In the context of therapeutic EV preparations, however, decoy-like activity may have two opposite meanings. It can be beneficial if the therapeutic goal is to neutralize a harmful ligand or inflammatory signal. But it can be counterproductive if vesicles bind receptors, occupy uptake routes, or accumulate in non-target cells without delivering the desired therapeutic signal. Therefore, the same concept—EVs acting as receptor- or ligand-interacting particles—may be either useful or harmful depending on disease context and product design.

### 5.5. Off-Target Uptake and Dilution of the Therapeutic Dose

Not all non-therapeutic EVs should be considered a distinct functional class. In particular, off-target uptake describes the fate of EVs rather than an intrinsic functional category. EVs carrying potentially therapeutic cargo may fail to contribute to the intended response simply because they are diverted to non-target cells or tissues. Thus, off-target distribution represents an important mechanism of therapeutic inefficiency that can affect the functional contribution of otherwise active EV subpopulations [101]. Surface composition is central to this process. EV surface molecules mediate interactions with extracellular matrix components, soluble proteins, and recipient-cell receptors. EV subpopulations with different adhesion profiles may have different biodistribution and different therapeutic relevance. A vesicle fraction with the correct cargo but the wrong surface profile may never reach or enter the intended target cell.

A vesicle fraction with the correct cargo but the inappropriate surface profile may never reach or enter the intended target cell, because EV uptake is strongly governed by surface-mediated interactions and recipient-cell recognition [109]. This provides an important explanation for why bulk EV preparations may produce inconsistent outcomes. The final effect is not determined only by what EVs carry, but also by where they go, which cells bind them, whether they are internalized, and whether their cargo becomes functionally available. In this sense, EV function is a product of both cargo identity and delivery competence.

Importantly, the functional diversity described above is observed even among EVs derived from the same nominal cellular source. Several studies now support the idea that a single donor-cell source can secrete functionally diverse EV subpopulations. Van de Wakker et al. showed that size-separated EV subpopulations from the same source differed in proteome and functional capacity, with some fractions being active and others showing little or no effect [110]. Wang et al. demonstrated that apical and basolateral EVs can differ in cargo and function, showing that release polarity contributes to EV diversity [98]. Liangsupree et al. further showed that marker-defined EV subpopulations can differ in cargo and antibody-binding capacity, supporting the use of surface markers to resolve EV heterogeneity beyond bulk analysis [111]. Taken together, these studies support a model in which the biological outcome of a bulk EV isolate reflects the combined contribution of multiple co-existing subpopulations. Some vesicles may be beneficial, some neutral, and some may reduce or redirect the intended response. Therefore, the therapeutic goal should be to identify, enrich, and characterize the most effective EV subtypes while minimizing neutral, off-target, or counterproductive fractions. This concept also explains why EV preparations derived from the same nominal cell type can have variable activity across studies. Differences in donor cell state, culture conditions, passage number, priming, isolation method, storage, and fractionation can shift the relative abundance of active versus inactive vesicles. Thus, reproducibility requires not only standardizing the cell source, but also controlling the functional composition of the EV product.

Taken together, these studies demonstrate that a single nominal cell source can generate EV populations with distinct molecular and functional properties. Importantly, this variability may arise not only from differences between cellular sources, but also from differences in donor cell state, culture conditions, passage number, priming, isolation method, storage, and fractionation. Consequently, reproducibility cannot be achieved by standardizing the parental cell source alone; it also requires control and characterization of the composition and functional properties of the resulting EV product.

These observations raise a broader therapeutic question: how should functionally distinct EV subpopulations be conceptualized in the context of therapeutic development?

### 5.6. Functional Classification as a Therapeutic Framework

From a therapeutic perspective, EV subpopulations could be organized into three functional classes. The first class includes therapeutically functional EVs, which contain disease-relevant cargo, reach the intended cells, and induce the desired biological response. The second class includes neutral EVs, which are present in the product but do not meaningfully contribute to the therapeutic effect in a given context. The third class includes antagonistic/competitive or decoy-like EVs, which may bind receptors, compete for uptake, alter biodistribution, block signaling, or be captured by off-target cells.

This framework is not meant to replace formal EV nomenclature, but to complement it. Biogenesis, size, density, morphology, and marker expression remain important for characterization. However, therapeutic development requires an additional functional layer. A vesicle’s value depends not only on whether it is an exosome-like or microvesicle-like particle, but also on the effect it exerts on the intended therapeutic task.

This approach is especially relevant in neurological diseases. Different neurological conditions require different mechanisms of action. A vesicle population optimized for anti-inflammatory signaling may not be optimal for axonal regeneration. EVs designed to modulate microglia may not efficiently target neurons or brain endothelial cells. Vesicles carrying neurotrophic cargo may be ineffective if they are trapped in peripheral organs or internalized by non-target immune cells. Therefore, EV products should be classified and optimized according to disease-relevant function, such as neuro-immunomodulatory EVs, neurotrophic EVs, BBB-targeting EVs, or pro-regenerative EVs, rather than only by their parental cell source.

## 6. Translational Implications

The functional heterogeneity of EVs has direct consequences for clinical translation. First, the inherent difficulty in comprehensively characterizing an EV mixture limits the utility of parameters such as particle number, total protein amount, or cargo content for therapeutic dose normalization. These parameters may correlate weakly with the functional potency of EVs. Dose normalization based on particle number possibly results in overestimation of effective EV dose due to the inclusion of non-functional vesicles or non-vesicular particles. Similarly, total protein amount may be skewed by non-vesicular proteins that co-purify during EV isolation procedures. Cargo content-based normalization does not account for differences in material packing among individual vesicles or for potential synergistic interactions between not assessed components. Consequently, if only a subset of vesicles within a preparation contributes to its functional activity, potency assays should assess biological activity rather than rely solely on particle concentration, protein content or cargo content.

Functional heterogeneity further highlights the need for detailed EV characterization based on subpopulation analysis, including size, density, morphology, surface markers, cargo, and uptake behavior. It also supports enrichment strategies aimed at increasing the proportion of active vesicles and reducing neutral or counterproductive fractions [95].

A separate but related challenge is the lack of standardized approaches to EV therapeutic dose normalisation. Differences in the parameters used across individual studies to normalize EV therapeutic dose and assess functional activity limit the direct comparability of results and hinder the accurate evaluation of the relationship between EV functional activity and the administered dose. Overall, EV preparations should be viewed as heterogeneous functional ecosystems rather than uniform therapeutic products. Once heterogeneity is interpreted in functional terms, the next translational challenge is to control the relative abundance and properties of these subpopulations rather than simply increase total EV yield.

## 7. From Heterogeneity to Controllable Product Design

At the donor-cell level, the first and arguably most important step in reducing MSC-EVs heterogeneity is the careful selection of the MSC source, as tissue origin substantially affects EV yield, cargo composition, and biological activity. Accordingly, source selection should precede any additional manipulation, particularly when designing EV-based therapeutics for neurological diseases. One proposed strategy to improve batch-to-batch consistency is the use of clonal MSC populations or immortalized MSC lines with stable genomic and functional characteristics. Although this approach may provide a scalable and relatively uniform source of vesicles, it does not fully eliminate heterogeneity and raises concerns related to long-term genomic and epigenetic stability, since extended culture and genetic manipulation may induce chromosomal abnormalities, transcriptional drift, or epigenetic reprogramming.

Among upstream approaches, genetic modification is the most precise strategy for enriching MSC-EVs with defined therapeutic cargo. This method has been used to enhance EV-mediated neuroprotection by overexpressing miRNAs, neurotrophic factors, or targeting ligands relevant to specific pathological processes such as neuroinflammation, impaired neurogenesis, or axonal degeneration [112]. For example, Yu et al. transduced bone marrow-derived MSCs with miR-29b using a lentiviral system demonstrating that administration of EVs enriched in miR-29b increased spinal cord miR-29b levels and improved outcomes in a rat model of spinal cord injury, including increased numbers of NF-220- and GAP-43-positive neurons [113]. Similarly, Huong et al. utilized lipofectamine-mediated modification of rat bone marrow MSCs with miR-126 and evinced that exosomes enriched in miR-126 reduced lesion area, inhibited neural apoptosis, and promoted angiogenesis, neurogenesis, and functional recovery after spinal cord injury [114]. Genetic engineering has also been applied to increase the secretion of neuroprotective proteins. Ahn et al. overexpressed brain-derived neurotrophic factor (BDNF) in irradiated immortalized bone marrow MSCs using lentiviral transduction and reported markedly enhanced BDNF secretion, accompanied by improved protection against oxygen-glucose deprivation-induced cytotoxicity, oxidative stress, and cell death in vitro, as well as reduced brain injury, apoptosis, astrogliosis, and inflammation in a severe neonatal hypoxic–ischemic brain injury model [115]. In principle, non-viral approaches, such as electroporation-mediated gene delivery, could also be used to express neuroprotective genes in MSCs, thereby generating EVs enriched in therapeutic factors. However, direct experimental evidence for this strategy remains far more limited than for viral engineering.

### Engineering Therapeutic Extracellular Vesicles for Clinical Translation

Engineering strategies have the potential to shift extracellular vesicles from inherently heterogeneous biological products toward more defined and reproducible therapeutic platforms at both the vesicle and cell level. Approaches such as surface functionalization, membrane engineering, and hybridization with synthetic nanoparticles can introduce more controlled physicochemical properties, whereas genetic engineering of donor cells can promote selective sorting of therapeutic cargos into EVs [116]. This can substantially improve therapeutic potency by increasing the amount and specificity of functional cargo delivered to target cells. Bio-orthogonal click chemistry seems to be an interesting approach which allows modification of the surface of EVs under relatively mild conditions, potentially preserving vesicle integrity while enabling the attachment of therapeutic molecules [117].

Engineering EVs with targeting ligands can help address the nonspecific uptake and clearance of EVs, particularly by the liver and spleen [109,118]. Enriching their surfaces with targeting peptides, antibodies or other recognition elements can enhance tissue accumulation and cellular internalization. Bio-orthogonal glycoengineering appears to be interesting because it can generate selective chemical handles on tumor cells and their associated EVs, enabling targeted therapeutic interactions even when conventional receptor–ligand targeting is limited by receptor heterogeneity [119].

Engineering may also improve reproducibility, but only if it is coupled with standardized manufacturing and quality control. Controlled cell culture conditions, defined engineering steps, scalable purification, and quantitative characterization of EV size, surface markers, cargo loading, purity, and functional activity could reduce batch-to-batch variation [116].

Overall, engineering is likely to reshape EV heterogeneity by moving from uncontrolled natural variation toward rationally specified vesicle populations with defined cargo, surface properties, and biological functions.

## 8. Priming as a Strategy for Functional Control of EVs

Among upstream strategies, priming is particularly relevant because it exploits the responsiveness of parental cells to reshape EV output without requiring direct modification of individual vesicles. Since EVs reflect the physiological and activation state of their donor cells, changes in the cellular microenvironment can alter EV cargo, surface composition, density distribution, uptake properties, and biological activity [114,120,121]. Priming can therefore be used to modulate the functional composition of MSC-EV preparations and potentially increase the relative abundance of vesicles with therapeutically desirable properties.

A variety of priming stimuli have been investigated to enhance the therapeutic activity of MSC-EVs, particularly in models of neurological injury and disease. These approaches include exposure of parental MSCs to inflammatory cytokines, hypoxic conditions, pharmacological agents, or disease-relevant microenvironments, each of which can induce distinct changes in EV cargo and biological activity [60,122]. The resulting EV preparations have been evaluated for effects such as neuroprotection, immunomodulation, angiogenesis, neuronal survival, and tissue regeneration across different neurological disease models (Table 2).

Importantly, priming should not be interpreted as a strategy that eliminates EV heterogeneity. Rather, it may reshape the functional composition of the EV population by altering the relative abundance and properties of different subpopulations. Consequently, the effects of priming should be evaluated not only by changes in EV cargo or physicochemical characteristics, but also by disease-relevant functional assays demonstrating whether the intended therapeutic phenotype has been achieved.

Across multiple disease models, different priming regimens consistently reshape EV behavior, underscoring the idea that EV identity is defined not by origin alone, but by conditioning state. In models of stroke and focal cerebral ischemia, human BM-MSC-derived sEVs preconditioned in 1% O_2_ for 48 h improved microvascular length and branching density in ischemic tissue and enhanced restorative effects compared with EVs from normoxic MSCs, indicative of a distinct angiogenic EV phenotype [36]. In rodent spinal cord injury (SCI) models, hypoxia-preconditioned rat BM-MSC EVs increased functional recovery, promoted neuronal survival, reduced oxidative stress, and limited lesion size, demonstrating a robust neuroprotective activity profile [33]. Similar results were obtained with BM-MSC- or ADSC-derived EVs subjected to 1% O_2_ for 48 h, which upregulated miRNA or lncRNA cargo such as miR 216a 5p, miR 146a 5p, or LncGm37494 and shifted microglial polarization from M1 to M2 while promoting functional recovery [32,34,35,38]. In parallel, hypoxia-preconditioned ASC-derived EVs carrying circEcp1 improved cognition and reduced neuronal damage in Alzheimer’s disease models by modulating microglial phenotype via the circEcp1–miR 770 5p–TREM2 axis [37]. In traumatic brain injury, BDNF or 3D conditioned MSC-derived EVs increased expression of BDNF, VEGF, IL 10, and NGF and improved sensorimotor and cognitive recovery, partly by promoting endogenous angiogenesis and neurogenesis [45,51]. In ischemic limb models, MSC EVs released from MSCs cultured with NO-releasing polymers enhanced VEGF and miR 126 levels and improved limb function, illustrating a pro-angiogenic EV phenotype engineered at the donor level [49]. In SCI, 4D or chemically primed (e.g., curcumin) MSC EVs polarized macrophages/microglia toward an M2 phenotype, reduced neuroinflammation and neuronal loss, and supported axonal regeneration, generating “regenerative” EVs enriched with IGFBP2, EGFR, or other repair-related molecules [39,48,50]. In other neurodegenerative contexts, IFN γ-primed, IL 23-primed, or H2S-preconditioned MSC EVs upregulated specific miRNA sets and immunomodulatory molecules and suppressed neuroinflammatory signaling, further highlighting the versatility of priming derived EV phenotypes [41,42,44,47].

Among the various priming strategies evaluated across disease models, hypoxia preconditioning and inflammatory cytokine-based priming (e.g., IFN γ, TNF α, IL 1β, IL 4, IL 10, IL 23) are currently the most widely used and best-characterized approaches [32,36,38,41,42,44,46]. Hypoxia, typically applied at 1% O_2_ for 48 h, is particularly prevalent in spinal cord injury, stroke, and ischemic tissue models, where it consistently enhances the neuroprotective, anti-inflammatory, and pro-angiogenic profiles of MSC-derived EVs [33,34,35,38]. Moreover, inflammatory priming is favored in neuroimmunomodulatory settings, including Alzheimer’s disease, ALS, and TBI, where it upregulates immunoregulatory molecules and shifts microglial/astrocytic responses toward a repair-oriented phenotype [41,42,44,46]. Together, these approaches represent the dominant paradigm in current EV priming research, reflecting their relatively straightforward implementation, good safety profile, and strong alignment with disease-relevant microenvironments.

In some cases, priming may also result in the incorporation of the conditioning compound into newly secreted EVs; however, evidence for this mechanism remains limited, with curcumin representing one of the better-documented examples [45].

Another emerging upstream concept is metabolic engineering of donor cells. In contrast to genetic engineering, this approach is aimed primarily at modifying cell and EV surface chemistry rather than enriching EV cargo with neuroprotective biomolecules. Most commonly, metabolic engineering targets glycan biosynthesis pathways in donor cells to generate EVs with altered surface composition, improved functionalization capacity, or enhanced targeting potential. Thus, in the context of neurological disease, metabolic engineering may be especially valuable for improving brain tropism, BBB crossing, and selective uptake by neural cells, rather than for directly increasing the content of therapeutic proteins or RNAs [123].

At the post-isolation level, isolated EVs can be engineered to overcome some of the limitations imposed by donor-cell heterogeneity. These downstream approaches can be divided into surface modification and cargo-loading strategies. Surface engineering includes peptide-based functionalization, glycan modification, phospholipid modification, click chemistry, and hybridization with heterologous EVs or liposomes, all of which may increase targeting specificity, including targeting toward the central nervous system [124,125]. Cargo loading of isolated EVs may be achieved passively by simple incubation with the desired compound, which is most suitable for low-molecular-weight hydrophobic agents such as curcumin or doxorubicin [14,126]. Active loading methods include electroporation, sonication, extrusion, microfluidics, freeze–thaw cycles, membrane permeabilization with agents such as saponin, and hybridization with other vesicular systems [14,127,128,129]. In a comparative study of catalase loading into EVs, loading efficiency increased in the order of incubation at room temperature without saponin < incubation with saponin < freeze–thaw cycling < sonication approximately equal to extrusion, illustrating how strongly the method of engineering can affect the amount of incorporated cargo [14]. However, even these post-isolation strategies do not fully solve the problem of heterogeneity, because not all EVs within a given preparation are equally modifiable or equally loaded, which may further contribute to functional dispersion within the final product [120].

Hybrid EVs are designed to combine the biological advantages of native EVs with the controllable physicochemical and cargo-delivery properties of synthetic nanoparticles. Given that the intrinsic composition of native EVs is difficult to control, incorporating a synthetic component may improve control over selected attributes, including particle size, membrane properties, and therapeutic cargo, while enabling more precise engineering of delivery functions [116,130].

Neurological research illustrates this potential. Jiang et al. showed that EV–liposome hybridization enables brain-targeted delivery of defined nucleic acid cargo, thereby potentially reducing reliance on variable endogenous EV contents [131]. Aliakbari et al. combined MSC-derived exosomes with baicalein- or oleuropein-loaded liposomes to reduce α-synuclein aggregation and neurotoxicity in vitro, whereas Chakravarty et al. reported improved stability and enhanced uptake of angiopep-2-decorated hybrids by LRP-1-expressing cells [132,133]. EV–EV hybrids, also referred to as dual-source hybrid EVs, represent another hybridization strategy. Wang et al. generated MSC–astrocyte hybrid EVs with more consistent size profiles and combined neuroprotective, immunomodulatory, and brain cell-targeting properties, although molecular homogeneity at the single-particle level was not established [128].

Collectively, these studies demonstrate improved control over selected formulation and delivery properties, which may mitigate some therapeutic limitations associated with EV heterogeneity, rather than a direct reduction in their biological heterogeneity. Hybridization does not eliminate source-dependent variability and may introduce new sources of heterogeneity, including incomplete fusion, uneven cargo loading, and cargo leakage. Single-particle analyses have revealed heterogeneous cargo loading and fusion outcomes, highlighting the need to characterize individual particles rather than rely solely on bulk measurements [134,135]. Hybridization should therefore be considered a strategy for reducing heterogeneity in specific physicochemical and cargo-related attributes, while any reduction in biological heterogeneity requires direct experimental confirmation. Its potential benefits must also be weighed against additional complexity associated with manufacturing, purification, characterization, and regulatory requirements.

Taken together, strategies for controlling MSC-EV heterogeneity should be viewed as a hierarchical manufacturing framework rather than as isolated interventions. Source selection and controlled upstream conditioning can shape the overall functional composition of EV preparations, while parental-cell engineering and downstream modification provide additional means of tuning cargo and targeting properties. Importantly, none of these approaches is expected to eliminate vesicle-to-vesicle variability completely; rather, their value lies in shifting heterogeneous EV populations toward a more predictable, disease-relevant functional profile.

## 9. Future Directions

A major future direction in MSC-EV research is to move beyond the concept of bulk EV preparations and address their intrinsic heterogeneity in a more function-oriented way. A key knowledge gap remains the extent to which multiple EV subpopulations coexist, interact with one another, and are differentially taken up by recipient cells. This is particularly important in the context of the central nervous system, where EVs must reach specific anatomical locations and act in a highly specialized microenvironment without disturbing neuroimmune homeostasis. A further challenge is to determine whether morphological EV subtypes are biologically meaningful, as the relationship between morphology and function remains largely unknown [136].

### Single-Vesicle Technologies

Addressing these questions requires analytical methods that resolve EVs at the level at which heterogeneity actually exists: the individual vesicle and defined subpopulation. Several techniques are currently available for the analysis of extracellular vesicles, including nano-flow cytometry, ExoView, nanoparticle tracking analysis (NTA), super-resolution microscopy, and EV sequencing [68,103,104,105,107,108]. These approaches provide complementary information on EV size, surface marker expression, morphology, molecular cargo, and subpopulation structure, and may help establish links between individual EV characteristics and biological activity.

Nano-flow cytometry enables EV characterisation using a low-flow system combined with the simultaneous detection of light scattering and fluorescence signals [137,138]. This approach allows multiplex and multicolour in situ analysis of individual EVs, including the assessment of vesicle size and surface marker expression [139]. A prime example of the use of nano-flow cytometry based on single-vesicle analysis is the ability to detect Alzheimer’s disease in its early stages. The researchers additionally used EV-CATCH technology for the controlled capture and release of extracellular vesicles from serum, and then profiled four key biomarkers of Alzheimer’s disease, demonstrating the high diagnostic accuracy of this method in the early stages of the disease [140].

Another platform for single-EV analysis is ExoView, which enables protein profiling at the level of individual vesicles [138]. Similarly, single-molecule localisation microscopy allows the spatial localisation of EV cargo within individual vesicles [138]. This analysis revealed different biological profiles of EVs depending on the source of MSCs and whether they were cultured in 2D or 3D conditions. The CD81 marker was more prominent in EVs derived from BM-MSCs or in 3D cultures, while CD9 was more prominent in those derived from corneal MSCs and 2D cultures. Additionally, results from ExoView revealed a more heterogeneous protein profile of EVs from cells cultured in 3D compared to 2D [141].

Nanoparticle tracking analysis (NTA) measures the diffusion of individual EVs and provides information regarding particle concentration, size distribution, and biomarker-defined subpopulations through selective fluorescent labelling [141]. The Demet team used NTA to determine the size of the EVs derived from WJ-MSCs, which was 104.6 ± 1.6 nm [122].

Super-resolution microscopy enables the visualisation of individual EVs with high spatial precision. In particular, Single Extracellular Vesicle Nanoscopy (SEVEN) allows detailed characterisation of EV subpopulations and identification of disease-specific EV biomarkers [139]. One such method is the STORM (stochastic optical reconstruction microscopy) method. It involves examining the structure of EVs from WJ-MSCs. The study revealed an FWHM (full-width half-maximum) of 110.86 nm. The study also showed hollow, spherical vesicle structures with bilaterally symmetrical fluorescent signals along the membrane. The distance between the peaks of the opposing membrane signals was approximately 84 nm, corresponding to the typical intermembrane distance of intact vesicles [122].

Another emerging approach is EV sequencing, which enables RNA-based EV profiling and further demonstrates EV heterogeneity [139]. This method represents a promising direction for research on EVs and is likely to be increasingly used in the coming years in studies analyzing the heterogeneity and functionality of extracellular vesicles.

Furthermore, focusing on EV secretion at the single-cell level enables the investigation of EV secretion dynamics and may provide insights into cellular states and mechanisms of intercellular communication [138].

In this context, single-vesicle analysis is not only a characterization exercise; it becomes a means to test whether priming, engineering, and manufacturing changes genuinely enrich the intended functional EV subpopulations.

Future progress will also depend on more rational EV product design, in which priming may play a central role in obtaining more homogeneous, functionally consistent, and disease-relevant EV populations. Priming should therefore be considered not only as a method to enhance EV yield or potency, but also as a strategy to remodel cargo composition, surface phenotype, and bioactivity in a controlled manner. Comprehensive proteomic, RNA, and DNA characterization using emerging single-EV and low-input technologies may provide a more complete understanding of how priming reshapes EV composition and biological activity [68,103,104,105,107,108].

Disease-specific priming of MSCs may help align EV products with the pathological context and improve therapeutic specificity. However, priming conditions need to be carefully standardized, because differences in stimulus type, intensity, duration, and timing can profoundly alter EV composition and introduce unwanted variability. It will therefore be essential to identify priming strategies that reproducibly enhance desired functions while minimizing pro-inflammatory, pro-thrombotic, or other off-target effects.

Finally, consistency across the field will require standardization not only of EV isolation but also of priming strategies and readouts of biological activity. This is critical prior to clinical translation, where successful bench-to-bedside advancement will rely on robust methodologies for defining EV identity, potency, and safety. These advances should help establish whether EV morphology, cargo, and functional subtype can be systematically connected, and whether such information can be used to improve therapy.

## Figures and Tables

**Table 1:** Main advantages and limitations of EV-based therapies compared with stem cell-based approaches.

Therapy	Advantages	Limitations
EVs	lower immunogenicity (antibody production) [11]	difficult to upscale manufacturing and purification [12]
	better bioavailability, for example: crossing blood–brain barrier [13,14,15]	uncontrolled prolonged release [16]
	improved stability and activity (in comparison to naked compound) [14]	
	stable in short- and long-term storage (lyophilization) [17,18]	
	possible intranasal administration [15,19]	
	surface engineering [13,17,19]	
	engineering of cargo [9,12,18]	
	fewer off-target effects [13]	
Stem cells	longer therapeutic effect than EVs [3]	uncontrolled cell division and differentiation [3,20],
	adaptable/context-responsive secretory profile	variable activity depending on the microenvironment [21]
		genetic instability [22]
		possible strong immune response [23,24]
		risk of secondary infections [25]
		off-target cell accumulation after intravenous administration [26,27]
		limited possibility of off-the-shelf products [17]

**Table 2:** Summary of MSC priming methods for the generation of EVs with enhanced neuroprotective properties. SCI—spinal cord injury; AD—Alzheimer’s disease TBI—traumatic brain injury; ND—neurodegenerative diseases; HIBI—hypoxic–ischemic brain injury; SE—status epilepticus.

Priming Method	Disease Model	Source	Dose	Main Changes in EVs	Effect	References
**Hypoxia 1% O_2_, 48 h**	Stroke; focal cerebral ischemia	rBM-MSCs	0–250 µg/mL	-	increased microvascular length and branching density; enhanced angiogenic and restorative effects of hypoxia-preconditioned MSC-sEVs	[36]
**Hypoxia 1% O_2_, 48 h**	SCI	rBM-MSC	0.2 µg/µL, 3 times	-	promoted neuron survival and reduced oxidative stress in vitro and in vivo; functional recovery, promoted nerve survival, and decreased lesion area	[33]
**Hypoxia 1% O_2_, 48 h**	SCI	BM-MSCs	200 µg in 200 µL	miR-216a-5p upregulation	improved behavioral recovery by shifting microglia toward the M2 phenotype through miR-216a-5p-mediated suppression of TLR4/NF-κB and activation of PI3K/Akt	[34]
**Hypoxia 1% O_2_; 48 h**	SCI	ADSCs	200 µg/mL	LncGm37494 upregulation	enhanced SCI repair by reducing inflammatory factors, improving functional recovery, and promoting M1-to-M2 microglial polarization via the miR-130b-3p/LncGm37494/PPARγ axis	[35]
**Hypoxia 1% O_2_; 48 h**	SCI	BM-MSCs	100 µg, 3 consecutive days	miR-146a-5p upregulation	reduced spinal cord inflammation and modulated macrophage polarization via miR-146a-5p transfer	[32]
**Hypoxia 1% O_2_**	AD	mADSCs	1 × 10^9^ particles w 100 µL	circEcp1	promoted cognition and reduced neuronal damage via the circEcp1/miR-770-5p/TREM2 axis, promoting M1-to-M2 microglial polarization	[37]
**Hypoxia 1% O_2_, 48 h**	SCI	rBM-MSCs	100 µg, 3 consecutive days	miR-21 upregulation	more effectively improved motor recovery and reduced apoptosis and inflammation after SCI by delivering miR-21; promoted astrocyte transition from the A1 to the A2 phenotype	[38]
**Hypoxia 1% O_2_, 24 h**	SCI	mBM-MSCs	1 × 10^12^ particles	miR-615-3p upregulation	hypoxia-conditioned BMSC-derived exosomes exerted neuroprotective effects via the miR-615-3p/PDE4C axis.	[39]
**Infarcted tissue extracts, 24 h**	Stroke	UCMSCs	80 µg/injection; 3 injections	139 miRNAs up-regulated, including hsa-miR-211-3p, hsa-miR-181a-5p, hsa- miR-148a-3p	Promoted vascular remodeling and recovery of neurological function	[40]
**TNFα** **(20 ng/mL) and IFNγ (25 ng/mL), 48 h**	AD	BMMSCs	30 µg, in two separate application, intranasal mice	-	MSC-EV exerted anti-inflammatory effects via COX-2 and IDO upregulation in in vitro and in vivo models	[41]
**IFN-γ 10 ng/mL serum free; 24 h**	ALS	BM-MSCs	Equivalent MSCs (EVs donor) and recipient cells	miR-467f; miR-466q, miR-466m-5p, miR-466i-3p upregulated	reduced expression of neuroinflammation markers	[42]
**IL-1 10 ng/mL, 24 h**	SE	hBM-MSCs	5 µg/mL	BDNF, VEGF, IL-10, NGF upregulation	suppressed LPS-induced astrogliosis and inflammatory responses of astrocytes; diminished the inflammatory response and stimulated the cognitive performance	[43]
**IL-23 20 ng/mL; 24 h**	ND	mBM-MSCs	5 × 10^10^ particles/mL in 2 μL	117 differently expressed miRNAs	enhanced restoration of retinal neural function in both in vivo and in vitro models; transition of M1 to M2 microglial polarization promoted by a set of anti-inflammatory miRNAs in MSC-exo	[44]
**BDNF, 30 ng/mL, 48 h, serum free**	TBI	rBM-MSCs	50 µg/mL in vitro, 100 µg/mL in vivo	miR-216a-5p upregulation	stimulated recovery of sensorimotor function and spatial learning ability; hindered inflammation and promoted neuronal regeneration in vivo and in vitro models	[45]
**Combinatorial: hypoxia (2% O_2_) and inflammatory signals 2% IFN-γ 50 ng/µL, TNF-α 10 ng/µL, and pH 6.2**	NDs	ADSCs	10 µL (0.5–2 × 10^10^)	-	increased EV number (particles and protein); promoted immunomodulatory properties	[46]
**H_2_S (NaHS),**	HIBI	mBM-MSCs	5 × 10^9^/mice 10 µg/5 × 10^5^	miR-7b-5p upregulation	reduced brain tissue loss, lowered pro-inflammatory mediators, promoted an anti-inflammatory state, and preserved long-term cognitive and memory functions, compared with non-primed EVs; associated with increased miR-7b-5p in EVs	[47]
**Curcumin 20 µM, 48 h**	SCI	mUC-MSCs	1 µg in 100 µL; four times, every 7 days	-	Cur-EVs/EVs improved locomotor function, attenuated inflammation, and enhanced axonal regeneration in mice, with Cur-EVs showing greater efficacy through promotion of M1-to-M2 macrophage polarization	[48]
**NO-releasing polymer**	Ischemic limb	hP-MSCs	20 µg in vitro, 10 µg in vivo	VEGF, miR-126 upregulation	improved angiogenic properties and ameliorated limb function in a murine model, upregulated VEGF and miR-126	[49]
**4D**	SCI	hUC-MSCs	500 µg, single injections in vivo; 40 µg in vitro	IGFBP2, EGFR upregulation	polarized macrophages/ microglia from the pro-inflammatory M1 to pro-regenerative M2 phenotype via the EGFR/STAT3 pathway both in in vitro and in vivo models; decreased neuroinflammation and neuronal loss at the spinal cord injury site	[50]
**3D collagen scaffold; 48 h**	TBI	hMSCs	100 µg (~3 × 10^9^ particles; 2 × 10^6^ MSCs)	-	3D-derived hMSC exosomes improved post-TBI cognitive and functional recovery more effectively than 2D-derived exosomes, partly via angiogenesis, neurogenesis, and reduced neuroinflammation	[51]
**3D bioprinted models; 48 h for 21 days**	TBI	ADSCs	2 × 10^9^ in vitro; 1 × 10^10^ in vivo	GAS6 (growth arrest-specific protein 6) upregulation	reduced neuroinflammation and brain tissue loss while accelerating motor function recovery in TBI mice by 3D-sEVs; shifted pro-inflammatory microglia toward an anti-inflammatory polarization state	[52]

## Data Availability

No new data were created or analyzed in this study. Data sharing is not applicable to this article.

## Abbreviations

The following abbreviations are used in this manuscript:

AD	Alzheimer’s disease
ALS	amyotrophic lateral sclerosis
ASC	adipose-derived stem cells
BDNF	brain-derived neurotrophic factor
Cryo-TEM	cryo-electron microscopy
DopaEVs	dopamine-modified EVs
EMA	European Medicines Agency
EVs	extracellular vesicles
GAS6	growth arrest-specific protein 6
ICTRP	International Clinical Trials Registry Platform
ILV	intraluminal vesicle
MitEVs	EVs carrying mitochondria
MSC-EVs	mesenchymal stem cell-derived EVs
MSCs	mesenchymal stem/stromal cells
NO	nitric oxide
NTA	nanoparticle tracking analysis
PD	Parkinson’s disease
PKD2	phospholipase D2
RMEVs	EVs derived from regenerating muscle
ROS	reactive oxygen species
SCI	spinal cord injury
SEC	size-exclusion chromatography
SeEVs	selenium nanoparticle-coated EVs
SEVEN	Single Extracellular Vesicle Nanoscopy
STORM	stochastic optical reconstruction microscopy
TBI	traumatic brain injury
